# Effects of chin tuck against resistance exercise on post-stroke dysphagia rehabilitation: A systematic review and meta-analysis

**DOI:** 10.3389/fneur.2022.1109140

**Published:** 2023-01-09

**Authors:** Jing Liu, Qiuyi Wang, Jing Tian, Wanqiong Zhou, Yitian Gao, Xuemei Chen, Wei Zhang, Yajing Gao, Lanshu Zhou

**Affiliations:** ^1^School of Nursing, Naval Medical University, Shanghai, China; ^2^Nursing School, Peking University, Beijing, China

**Keywords:** chin tuck against resistance exercise, stroke, rehabilitation, dysphagia, systematic review

## Abstract

**Background:**

Chin tuck against resistance (CTAR) exercise was introduced to substitute for the commonly used Shaker exercise for dysphagia rehabilitation. The effects of CTAR exercise in stroke survivors needs to be validated.

**Objective:**

To investigate the effects of Chin tuck against resistance (CTAR) exercise on the swallowing function and psychological condition in stroke survivors compared to no exercise intervention and the Shaker exercise.

**Materials and methods:**

The Cochrane Library, PubMed, Web of Science, EMBASE, CINAHL and four Chinese databases were searched for randomized controlled trails (RCTs) and quasi-RCTs from inception to February 2022.

**Results:**

After screened and assessed the methodological quality of the studies, nine studies with 548 stroke survivors were included in the systematic review. 8 studies were included in the meta-analysis using RevMan 5.4 software. The mean difference (MD) or standardized mean difference (SMD) with 95% confidence intervals (CIs) were calculated. The results revealed that CTAR exercise is effective in improving swallowing safety (MD, −1.43; 95% CI, −1.81 to −1.06; *P* < 0.0001) and oral intake ability (SMD, −1.82; 95% CI, −3.28 to −0.35; *P* = 0.01) compared with no exercise intervention, CTAR exercise is superior to Shaker exercise in improving swallowing safety (MD, −0.49; 95% CI, −0.83 to −0.16; *P* = 0.004). The psychological condition in CTAR group is significant better than the control group (MD, −5.72; 95% CI, −7.39 to −4.05; *P* < 0.00001) and Shaker group (MD, −2.20; 95% CI, −3.77 to −0.64; *P* = 0.006).

**Conclusions:**

Our findings support CTAR exercise as a superior therapeutic exercise for post-stroke dysphagia rehabilitation than Shaker exercise. More high-qualities RCTs from larger multicenter are needed to analysis the effects of CTAR exercise in patients with different type and phase of stroke and explore the optimal training dose.

## Introduction

Dysphagia is any difficulty during bolus transport from the oral cavity to the stomach in the swallowing process (1). It is one of the most common complications that affecting 37–78% stroke survivors (2, 3) and is strongly associated with a high risk of aspiration pneumonia, malnutrition, and increased mortality (4–6). For many patients, dysphagia resolves spontaneously within 14 days, but 50.9% of dysphagia persist at discharge, and 15% of patients still have dysphagia at 1 month of the onset of stroke, 11–50% still have dysphagia at 6 months (6–8). The residual functional deficits not only seriously affect the quality of life of stroke survivors, but also is a major cause of post-stroke depression and social isolation (9). Thus, exploring effective dysphagia rehabilitation methods is an essential concern of post-stroke care.

Therapeutic exercises that stimulating and strengthening the swallow-related muscles are strongly recommended for dysphagia rehabilitation (10). The suprahyoid muscle complex (SHM) is critical during the pharyngeal phase of swallowing as it controls the movement of the larynx, hyoid bone, and epiglottis to protect the airway, and the opening of upper esophageal sphincter to allow bolus transfer into the esophageal (11). For stroke survivors, based on the neuroplasticity principle, regular and repetitive resistance training can lead to the strength of swallowing muscles and may be effective on the recovery of sensorimotor control system of swallowing (12). Thus, SHM strengthening exercise has been a focus of research and practice in post-stroke dysphagia rehabilitation. The head-lift exercise (HLE), also called Shaker exercise, is the most commonly used SHM strengthening exercise that has been demonstrated to be effective in strengthen the SHM, reduce pyfiform sinus residue and increase upper esophageal sphincter opening in dysphagia (13, 14). It requires patients to lift their heads against gravity to look at their toes in a supine position (15). But Shaker exercise has some drawbacks. When patients raise their heads, the sternocleidomastoid muscle are inevitably activated, causing unnecessary muscle fatigue and physical effort (16). For elderly patients who are physically frail, repeated lifting of and holding their heads up is challenging. Several studies reported that participants showed a low compliance and felt frustrated (16–18). Therefore, chin tuck against resistance (CTAR) exercise was introduced as a new rehabilitative exercise that could substitute for Shaker exercise by Yoon et al. (19). For CTAR exercise, the patient is instructed by speech and language therapists to tuck their chin toward their manubrium sterni to squeeze an inflatable rubber ball that placed between their chin and chest while seated. Similar to Shaker exercise, CTAR exercise includes isometric and isokinetic tasks. The isokinetic task is the squeezing of the ball as hard as possible for successive repetitions, while the isometric task is the squeezing of the ball and sustaining the squeeze for a period of time (19). People can choose the appropriate resistance according to their physical condition. Several studies have been conducted to validate the biomechanics effects of CTAR exercise and compared with Shaker exercise using surface electromyography (sEMG), which demonstrate CTAR is effective in stimulating the SHM but there are inconsistent conclusions in the comparison of CTAR and Shaker exercise (20, 21). To our knowledge, a previous systematic review (22) summarized the applications of CTAR exercise, in which both healthy participants and patients with dysphagia were included. Due to the high heterogeneity and limit number of studies, they only performed a descriptive qualitative analysis that CTAR is more selective in the activation of the SHM than Shaker exercise. But whether the strength of SHM can elicit the improvement of swallowing function still needs to be verified, as post-stroke dysphagia is functional dysphagia caused by hemisphere damage rather than organic disorder. Additionally, we noticed that a series of RCTs that explore the effects of CTAR exercise in stroke survivors were reported recently. Therefore, the objective of this study was to included the newly published studies and performed a meta-analysis of the results on the effects of CTAR exercise in stroke survivors to provide a reliable evidence for the policy and practice development of post-stroke dysphagia rehabilitation.

## Materials and methods

This systematic review and meta-analysis was conducted based on the Cochrane Handbook for Systematic Reviews of Interventions (https://training.cochrane.org/handbook), and reported according to the Preferred Reporting Items for Systematic Reviews and Meta-Analyses statement (PRISMA) (23, 24). This review was previously registered on PROSPERO (CRD42021265975).

### Search strategy

We systematically searched the following electronic databases from inception to February 2022: Cochrane Library Cochrane Database of Systematic Reviews (CDSR), Cochrane Central Register of Controlled Trials (CENTRAL), PubMed, Embase, Web of Science (WOS), the Cumulative Index to Nursing and Allied Health Literature (CINAHL) for studies published in English, China Biology Medicine disc (CBM), China National Knowledge Infrastructure (CNKI), WanFang, and VIP database for studies published in Chinese. The following search terms were used: “chin tuck OR chin down OR CTAR” AND “stroke OR apoplexy OR cerebrovascular accident OR CVA OR brain vascular accident OR brain infarction OR cerebral infarction OR ischemic stroke OR hemorrhagic stroke” AND “dysphagia OR deglutition disorder OR swallowing disorder OR swallowing dysfunction OR impaired swallowing.” We also searched the reference lists of the included studies and Google Scholar to identify relevant studies.

### Eligibility criteria

Studies were included if they met the following criteria: (1) study design: a randomized controlled trial (RCT) or a quasi-RCT; (2) participants: adults diagnosed with post-stroke dysphagia that confirmed by a videofluoroscopy swallowing study (VFSS) or fiberoptic endoscopic evaluation of swallowing (FESS) or standardized dysphagia assessment instrument; (3) intervention and comparison: a comparison of CTAR exercise with no exercise intervention or with Shaker exercise; and (4) outcome measures: the primary outcome are the swallowing safety and oral intake ability as measured by standardized dysphagia assessment scale; the second outcome is psychological condition as measured by Self-Rating Depression Scale (SDS). There was no restriction on the language of publication.

Studies were excluded if they (1) were reviews, case reports, conference abstracts, expert opinion articles or peer-review publications or if (2) their full texts were not available or valid outcome data could not be extracted.

### Study selection and quality assessment

First, all searched studies were imported to NoteExpress 3.2.0 to delete duplicates. Then, two reviewers (L.J. and W.Q.Y.) independently completed the title and abstract screening to exclude irrelevant studies, followed by full-text screening according to the eligibility criteria. All reviewers were familiar with stroke rehabilitation and had taken an evidence-based training course.

The methodological quality of the included studies was assessed by two reviews (T.J. and Z.W. Q) independently. The Cochrane risk of bias tool for randomized controlled trials was used for 8 RCTs, in which 5 domains were examined: (a) selection bias, (b) performance bias, (c) detection bias, (d) attrition bias, and (e) reporting bias (25, 26). The risk of bias for each domain was reported as low, high, or unclear. The Joanna Briggs Institute (JBI) critical appraisal checklist was used for 1 quasi-experimental study (27). Discrepancies were resolved by discussion.

### Data extraction

Two reviewers (L.J. and W.Q.Y.) independently extracted the data using a predefined form, and the following data were collected: first author's name, publication year, sample size, participants' characteristics (age, type and phase of stroke), protocols for the intervention and control groups (device, frequency, repetition, and duration), outcome measures and results. The authors were contacted *via* email if incomplete data were provided for analysis.

### Statistical analysis

According to our objective, two comparisons were performed: CTAR exercise vs. no exercise intervention and CTAR exercise vs. Shaker exercise. Based on the Cochrane Handbook for Systematic Reviews of Interventions (version 6.3, 2022), meta-analysis consists of two stage. First, we calculated the mean change and standard difference from baseline to post-intervention in each group. The formulas were used if the standard difference was not presented (28):


(1) $$\begin{array}{l} Corr_{E}=\frac{SD_{E,baseline}^{2}+SD_{E,final}^{2}-SD_{E,change}^{2}}{2*SD_{E,baseline}*SD_{E,final}} \end{array}$$

(2) $$\begin{array}{l} SD_{E,change}\\ =\sqrt{SD_{E,baseline}^{2}+SD_{E,final}^{2}-\left(2*Corr_{E}*SD_{E,baseline}*SD_{E,final.}\right)} \end{array}$$

To ensure that different scales represented the same effect direction for outcome measurement, we chose the most commonly used scale to determine the effect direction, and the mean change in scale scores with different directions was multiplied by −1. Then, the meta-analysis was conducted using Review Manager software (RevMan, version 5.4). Mean difference (MD) (when all studies measure the outcome using the same scale) or standardized mean difference (SMD) (when studies measure the outcome using different scales) was calculated for each study and synthesized into a pooled effect size with 95% confidence interval (CI).

The heterogeneity across the studies was analyzed by statistical testing with *I*^2^. *I*^2^ values <40, 40–75%, and >75% were considered low, moderate, and high heterogeneity, respectively. Random effects models were used to perform meta-analyses. In this study, a *P*-value < 0.05 was considered statistically significant.

## Results

### Study selection

**Figure 1:**
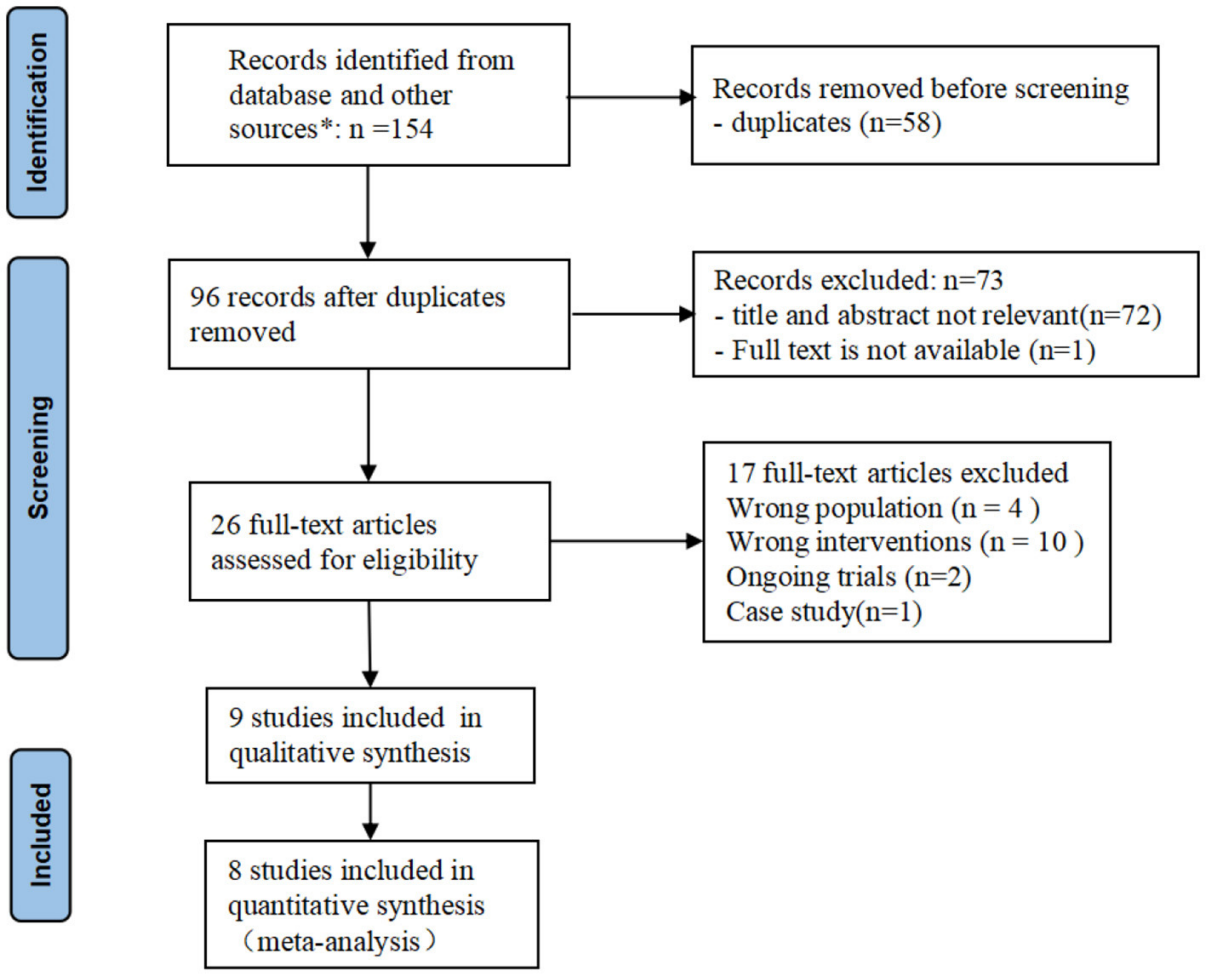
PRISMA flow diagram of study selection. *The number of records identified from each database searched: Cochrane Library: *n* = 11; Pubmed: *n* = 11; Web of science: *n* = 37; EMBASE: *n* = 27; CINAHL: *n* = 12; CBM: *n* = 2; CNKI: *n* = 35; WanFang: *n* = 13; VIP: *n* = 6.

A total of 154 articles were identified from databases, and 58 duplicates were removed using the duplicate finder tool in NoteExpress 3.4.0. Seventy-two studies were excluded after title and abstract screening, one study was excluded because full text was not available. Another 17 studies were excluded after reading the full text. One study from USA (29), one from Singapore (20), one from Turkey (30), and one from Netherlands (31) were excluded due to the wrong population. One study (32) from Greece was excluded because its intervention consisted of CTAR exercise and thermal-tactile stimulation so we could not evaluate the effects of CTAR exercise separately. Two ongoing RCTs (33, 34) from the UK and India were excluded. Finally, 9 studies were included in this systematic review, including 8 RCTs and one quasi-RCT. Five studies (35–39) came from China, three studies (40–42) came from South Korea, and one study (43) came from India. 8 studies was included in the meta-analysis as Lai's study (38) used the dysphagia screening tool rather than standardized assessment scale as outcome measures, which may limit the accuracy of the results. The PRISMA flow diagram shows the study selection process (see Figure 1).

### The risk of bias

**Figure 2:**
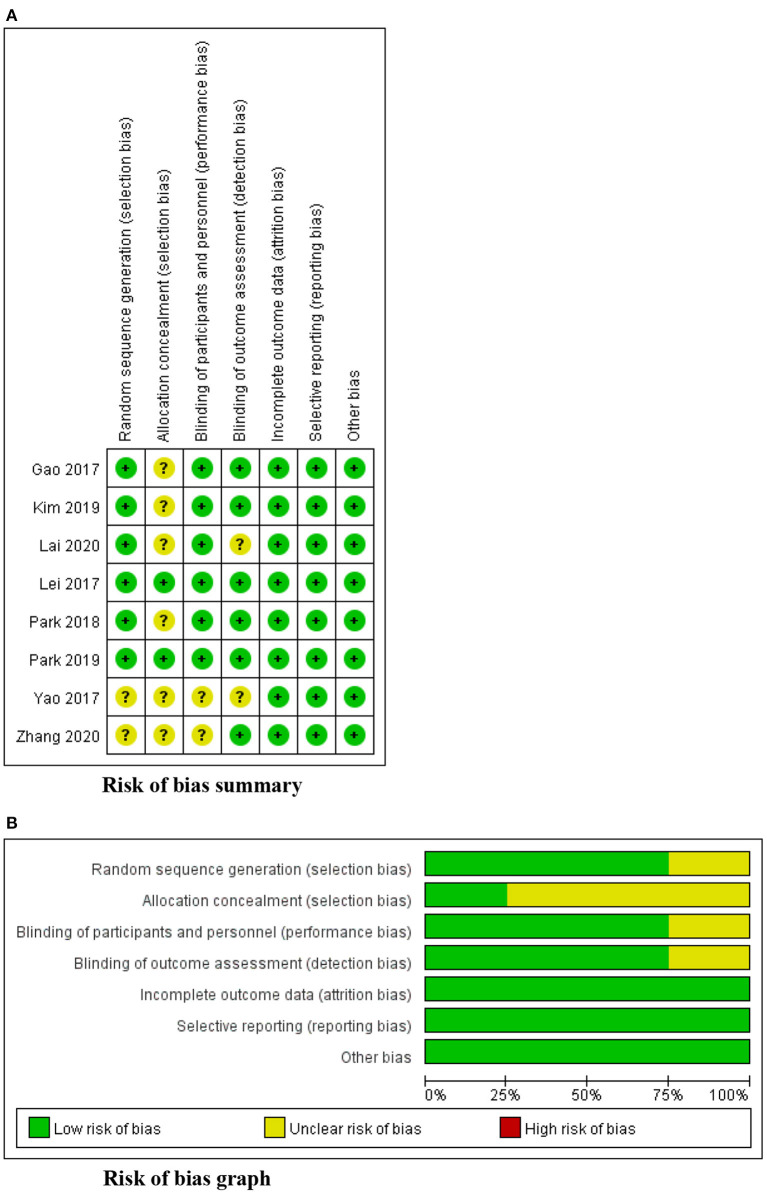
Risk of bias **(A)** summary **(B)** graph.

Figure 2 displays a summary of the risk of bias assessment for the 8 RCTs. Two studies provided insufficient details about their methods of randomization (35, 37). Only two studies reported the process of allocation concealment (36, 41). None of the 8 RCTs reported the blinding of participants and outcome assessments, but considering that participant blinding was impossible in exercise-based intervention, six studies were determined to have a low risk of performance bias (36–42), and the other two studies were considered to have a high risk of between-group sample contamination because of their unclear randomization and allocation (35, 37). For detection bias, six studies measured swallowing function using a standard evaluation scale based on VFSS (35, 36, 39–42); thus, they were considered to have a low risk of detection bias. No selective reporting and other bias were identified. The overall appraisal of one quasi-experimental study was “include.”

### Characteristics of the included studies

The characteristics of participants in the 9 included studies are shown in Table 1. A total of 548 participants were included, with sample sizes of each study ranging from 22 to 120. Five studies included both hemorrhagic and ischemic stroke patients (37, 40–43), and four studies included only ischemic stroke patients (35, 36, 38, 39). The post-stroke time varied from 4 days to 63 weeks.

**Table 1:** Characteristics of participants in the included studies.

**Author**	**Country**	**Study design**	**Sample size (IG/CG)**	**Mean age (year) IG/CG**	**Phase of stroke**	**Type of stroke**
Santhosh Priya (43)	India	Quasi-RCT	A:16/16	35–85	Acute	Hemorrhage, infarction
Gao and Zhang (39)	China	RCT	A:30/30	70.88/71.14	Acute	Hemorrhage, infarction
			B:30/30	70.88/71.12	Acute	
Yao et al. (37)	China	RCT	A:26/24	64.2/63.0	Acute	Hemorrhage, infarction
Lei and Guo (36)	China	RCT	A:30/30	70.8/71.14	Acute	Only infarction
			B:30/30	70.8/71.12	Acute	
Park et al. (42)	Korea	RCT	A:11/11	62.16/58.43	Chronic	Hemorrhage, infarction
Park et al. (41)	Korea	RCT	B:19/18	60.9/59.45	Chronic	Hemorrhage, infarction
Kim and Park (40)	Korea	RCT	A:12/13	63.5/65.2	Unclear	Hemorrhage, infarction
Zhang et al. (35)	China	RCT	A:40/40	72.46/73.36	Acute	Only infarction
			B:40/40	72.46/74.11	Acute	
Lai (38)	China	RCT	A:41/41	72.41/73.02	Both	Only infarction

The characteristics of the intervention are shown in Table 2. Of the included 9 studies, 3 studies were three-arm trials (35, 39, 42). Thus, a total of 12 datasets were analyzed. Eight of the datasets (411 patients) compared CTAR exercise combined with traditional dysphagia treatments (TDT) vs. only TDT (e.g., oral facial massage, thermal-tactile stimulation, and transcranial direct current stimulation). The other four datasets (137 patients) compared the effects of CTAR exercise vs. Shaker exercise.

**Table 2:** Characteristics of intervention and control group in the included studies.

**Author**	**CTAR device**	**IG**	**CG**	**Training parameters**	**Frequency**	**Follow up**	**Outcome measures**
Santhosh Priya (43)	Inflatable rubber ball	CTAR + TDT	A: TDT	Only isometric tasks: 10 s × 10	3 times/days, everyday	8 consecutive days	GUSS, FOIS
Gao and Zhang (39)	Inflatable rubber ball	CTAR + TDT	A: TDT	Only isokinetic tasks: 30 times × 3 (3 times/days)	Everyday	6 weeks	VFSS-PAS, SDS
			B: Shaker +TDT				
Yao et al. (37)	Inflatable rubber ball	CTAR + TDT	A: TDT	Only isokinetic tasks: 15 times × 3 (2 times/days)	5 days/weeks	4 weeks	FILS, WST
Lei and Guo (36)	Inflatable rubber ball	CTAR + TDT	A: TDT	Isokinetic tasks: 30 times × 3	Everyday	6 weeks	VFSS-PAS, SDS
			B: Shaker + TDT	Isometric tasks: 60 s × 3 (3 times/days)			
Park et al. (42)	Hand-held device	CTAR + TDT	A: TDT	Isokinetic tasks: 30 times	5 days/weeks	4 weeks	VFSS-PAS
				Isometric tasks: 60 s × 3			
Park et al. (41)	Game-based device	CTAR + TDT	B: Shaker + TDT	Isokinetic tasks: 30 times	5 days/weeks	4 weeks	VFSS-PAS, FOIS, patient feedback, drop-out rate
				Isometric tasks: 60 s × 3			
Kim and Park (40)	Hand-free device	CTAR + TDT	A: TDT	Isokinetic tasks: 30 times	5 days/weeks	6 weeks	VFSS-PAS, FOIS, NG tube removal
				Isometric tasks: 10 s × 3			
Zhang et al. (35)	Inflatable rubber ball	CTAR + TDT	A: TDT	Isokinetic tasks: 30 times × 3 (3 times/days)	Everyday	6 weeks	VFSS-PAS, SDS
			B: Shaker +TDT				
Lai (38)	Inflatable rubber ball	CTAR + TDT	A: TDT	Isometric tasks: 60 s × 30 (3 times/days)	Everyday	20 days	Water swallow test, SSA

IG, intervention group; CG, control group; TDT, traditional dysphagia treatment; A-CTAR + TDT vs. TDT, B-CTAR vs. Shaker; PAS, penetration-aspiration scale; FOIS, functional oral intake scale; FILS, Fujishima Ichiro food intake level scale; GUSS, Gugging and swallowing screen; WST, water swallow test; SSA, standardized swallowing assessment; SDS, self-rating depression scale.

Regarding CTAR intervention, six studies used an inflatable rubber ball placed between the chin and the sternum (35–39, 43), 1 study used a hand-held flexible resistance bar (42), 1 study used a modified hand-free resistance bar secured to a desk surface (40), and 1 study used a hand-held resistance bar connected to a game-based PC tablet screen (41). Only the game-based device could adjust the intensity of training resistance. For the training protocols, 3 studies involved only isokinetic tasks (35, 37, 39), 2 studies involved only isometric tasks (38, 43), and the other 4 studies involved both (36, 40–42). The isokinetic task was 1 set of 30 consecutive squeezes, while the isometric task was the holding of the squeeze from 10 to 60 s for 3 to 10 repetitions. The training frequency varied from 1×/day for 5 days/weeks to 3×/day for 7 days/weeks. The duration of treatment varied from 8 days to 6 weeks.

For outcome measures, swallowing function and psychological condition were primary outcomes. A total of 11 different scales were used to measure swallowing function: (1) Swallowing safety: Six studies used the Penetration-Aspiration Scale (PAS) based on VFSS (35, 36, 39–42). The PAS is a widely used standard assessment scale to evaluate swallowing safety, which includes 8 points to reflect the depth of bolus penetration into the airway and the airway response to invasion. Higher scores indicate higher levels of airway aspiration and greater aspiration severity (44). (2) Oral intake ability: Three studies used the Functional Oral Intake Scale (FOIS) (40, 41, 43), and 1 study used the Fujishima Ichiro Food Intake Level Scale (FILS) (37). The FOIS is a 7-point scale that describes the feeding performance of oral intake (45), while the FILS is a 10-point scale. For both scales, a score of 1 indicates total nasogastric (NG) feeding, and higher scores represent better oral intake ability. (3) dysphagia screening tool: Two studies used the Water Swallow Test (WST) (37, 38), 1 study used the Gugging Swallowing Screen (GUSS) (43), and 1 study used the Standardized Swallowing Assessment (SSA) (38). The WST, GUSS and SSA are all simple bedside tools for dysphagia screening that are commonly used to identify high-risk populations with dysphagia and estimate the severity of dysphagia (46). Regarding psychological condition, 3 studies used the Self-depression scale (35, 36, 39).

Other outcomes included the compliance of patients (41), the score on a self-reporting questionnaire of enjoyment and physical fatigue (41), the NG tube removal rate (40, 41) and the physiological changes during the detailed phases of the swallowing process (41, 42).

### Data synthesis

#### CTAR exercise vs. no exercise intervention

##### Swallowing safety as measured by PAS scores

**Figure 3:**
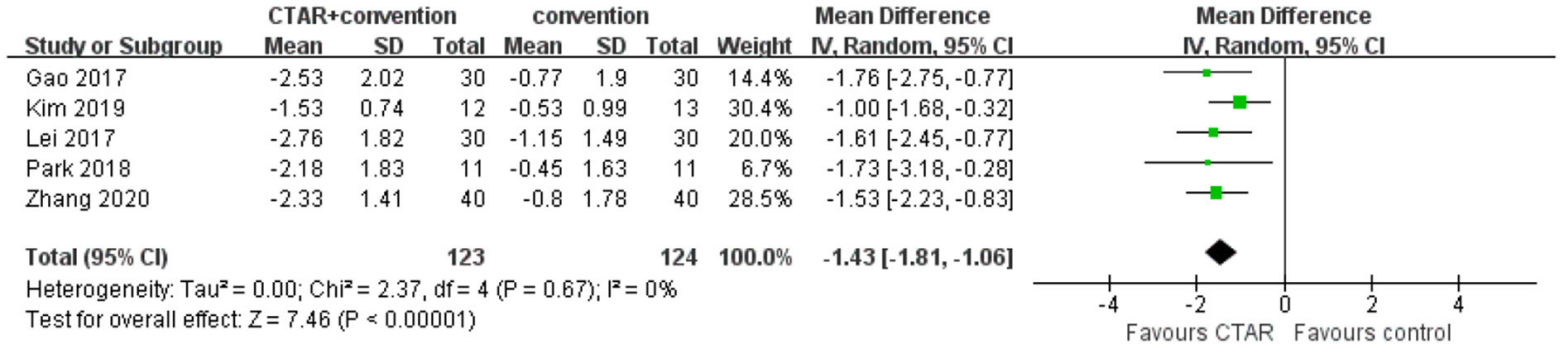
Pooled changes of swallowing safety in CTAR exercise vs. no exercise intervention.

The aggregated results of 5 studies (123 patients in CTAR group and 124 in the control group) showed that CTAR group had a significantly lower PAS score than the no exercise intervention group (MD, −1.43; 95% CI, −1.81 to −1.06; *P* < 0.00001; *I*^2^, 0%) (see Figure 3), which suggested that patients in CTAR group had better swallowing safety and a lower risk of aspiration. The studies were homogenous.

##### Oral intake ability as measured by FOIS or FILS scores

**Figure 4:**
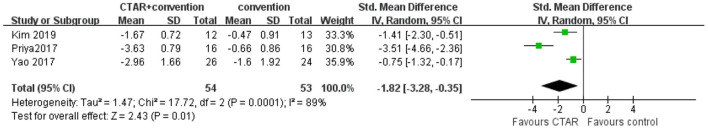
Pooled changes of oral intake ability in CTAR exercise vs. no exercise intervention.

The aggregated results of 3 studies (54 patients in CTAR group and 53 in the control group) showed a greater intervention-induced effect of oral intake ability in the CTAR group than that of the control group (SMD, −1.82; 95% CI, −3.28 to −0.35; *P* = 0.01; *I*^2^, 89%) (see Figure 4). The heterogeneity between studies was high.

##### Psychological condition as measured by SDS scores

**Figure 5:**
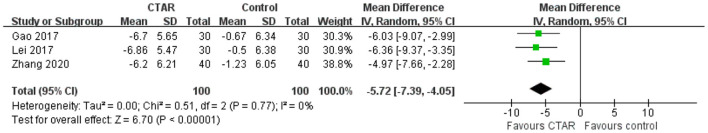
Pooled changes of psychological condition in CTAR exercise vs. no exercise intervention.

Three studies measured psychological condition using the SDS. The meta-analysis showed that the SDS scores in CTAR group were significantly lower than those in the control group (MD, −5.72; 95% CI, −7.39 to −4.05; *P* < 0.00001; *I*^2^ = 0%) (see Figure 5), which supported that the psychological condition of patients in CTAR group was significantly better than that in the control group. The studies were considered homogenous.

##### Other outcomes

Park et al. (42) evaluated the detailed phases of the complete swallowing process during a VFSS and found that CTAR group showed significantly better scores in the oral cavity and laryngeal elevation/epiglottic closure and less residue in the valleculae and pyriform sinuses. Kim et al. (40) reported that the rates of NG tube removal in CTAR and control groups were 25 and 15%, respectively.

#### CTAR exercise vs. Shaker exercise

##### Swallowing safety as measured by PAS scores

**Figure 6:**
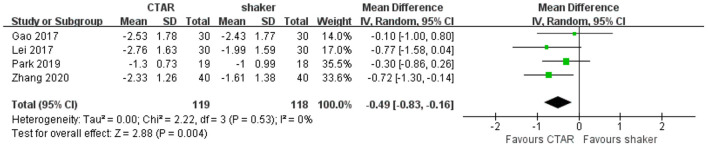
Pooled changes of swallowing safety in CTAR exercise vs. Shaker exercise.

Four studies (119 patients in CTAR group and 118 in Shaker group) used the PAS to assess swallowing safety and compared the effects of CTAR and shaker exercises. The aggregated results showed that the change scores of CTAR group was significantly greater than that of Shaker group (MD, −0.49; 95% CI, −0.83 to −0.16; *P* = 0.004; *I*^2^, 0%) (see Figure 6), which suggested that CTAR exercise was more effective in improving swallowing safety than Shaker exercise. The studies were considered homogenous.

##### Oral intake ability

One study measured oral intake ability using the FOIS, and the results showed no significant difference in FOIS scores between CTAR and shaker group.

##### Psychological condition as measured by SDS scores

**Figure 7:**
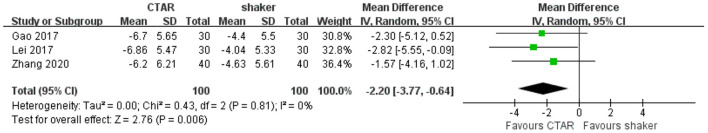
Pooled changes of psychological condition in CTAR exercise vs. Shaker exercise.

Three studies compared the SDS scores of CTAR and shaker groups. The aggregated results showed that the psychological condition of patients in CTAR group was significantly better than that of patients in Shaker group (MD, −2.20; 95% CI, −3.77 to −0.64 *P* = 0.006; *I*^2^ = 0%) (see Figure 7). The studies were considered homogenous.

##### Other outcomes

Park et al. (41) reported that CTAR group showed a significantly lower drop-out rate, better feedback in terms of motivation and interest/enjoyment, and lower physical fatigue than Shaker group.

## Discussion

The purpose of this systematic review was to investigate the effects of CTAR exercise on swallowing function and psychological condition of stroke survivors. Overall, the results showed a positive effect of CTAR exercise on improving swallowing safety, oral intake ability, and psychological condition compared with no exercise intervention. Compared with Shaker exercise, the results of the meta-analyses suggested that CTAR exercise was more effective in improving swallowing safety and psychological condition.

To our knowledge, this is the first systematic review and meta-analysis to examine the clinical effects of CTAR exercise in stroke survivors and confirm that CTAR exercise has superior effects than Shaker exercise in post-stroke dysphagia rehabilitation. The main results are consistent with a previous systematic review, which compared the effects of CTAR exercise in improving swallowing safety compared with no exercise intervention (47). But they did not compare oral intake ability and the effect of CTAR with Shaker exercise. Our findings demonstrated that strengthening exercise of SHM is not only fit for dysphagia that resulting from upper esophageal sphincter dysfunction, but also effective for rehabilitation of post-stroke dysphagia due to hemisphere damage. Previous studies that evaluated the biochemical changes of CTAR exercise reported that for instant muscle performance, CTAR exercise could exhibit significantly higher instant mean and max muscle fatigue of the SHM, with less stimulation of the sternocleidomastoid muscle than performing Shaker exercise (20). And after 8-week training, a significant greater anterior tongue pressure, and maximum mouth opening were observed in participants performing CTAR exercise compared to Shaker exercise (30, 31). Our findings, focus on the clinical effects of CTAR exercise in stroke survivors, supported that performing CTAR exercise to repetitive stimulating the SHM could translate into the biomechanics changes during the swallowing process and subsequently lead to increased swallowing safety and better recovery of oral intake ability, which is meaningful to avoid the risk of penetration-aspiration and improve the quality of life of stroke survivors. Additionally, another strength of our study is that we verify patients performing CTAR exercise have better psychological condition compared with no exercise intervention and Shaker exercise, which can be explained by two aspects. First, studies have shown that post-stroke depression is strongly associated with the severity of functional impairment, so better improvements in swallowing function can induce a positive effect on the patients' psychological condition (48). Second, in contrast to a considerable number of patients feeling frustrated with their failure to perform Shaker exercise, patients provided positive subjective feedback about CTAR exercise, as they could complete the exercise with suitable training intensity based on their physical condition, and CTAR exercise was more interesting and motivating, especially when the exercise was combined with computer games (19, 41). Therefore, we recommend CTAR exercise as the first choice rehabilitative exercise for stroke survivors with dysphagia. For patients who cannot sit, Shaker exercise can be used as an alternative.

However, we also found that the training protocol varied greatly in previous CTAR studies. Exercised-based therapy utilizes the neuroplasticity principle that repetition, intensity, frequency and duration are especially important to achieve muscular hypertrophy (12, 49). Incorrect dose prescription may cause insufficient or negative effects. Blair et al. (50) considered it is unlikely to establish an optimal dosing for a training exercise, the dosing should be determined by the patient's age, primary dysphagia etiology, comorbidities, and physical fitness level. But several studies have demonstrated that it takes at least 4 weeks of resistance training to induce physiological changes in stroke patients (13, 51, 52). Most studies included in this review conducted a 4- or 6-week intervention (35–37, 39–42), and the other 2 studies conducted interventions for only 8 and 20 days (38, 43). For the repetitions, 30 consecutive repetitions for the isokinetic exercise and 3 sustained 60-s squeezes for the isometric exercise were adopted by most studies, which was consistent with the recommended dose for Shaker exercise (49). However, 2 studies performed only isometric tasks (38, 43), while the other 3 studies performed only isokinetic tasks (35, 37, 39). The training frequency also varied from 1×/day for 5 days/weeks to 3×/day for 7 days/weeks. Therefore, future research should continue to explore the relationship between the training dose and the training efficacy and establish universal standard parameters for CTAR exercise to maximize patient benefits.

This study has several limitations. First, there were heterogeneity between the included studies in terms of the patient conditions, type of stroke, and time post-stroke. The meta-analysis of oral intake ability and the overall severity of dysphagia showed substantial statistical heterogeneity, which may be due to the different measurement scales used in the studies. As Egger's publication bias test is known to have limit efficiency for meta-analysis that involving <10 studies, we did not conduct the test. Second, some studies had a high risk of bias in terms of randomization and allocation, which may limit the quality of the evidence. Third, most studies included in this review were from South Korea and China, which may limit the generalization of our findings. But this does not mean that no CTAR studies were performed in other countries. As CTAR exercise was proposed in 2014, most studies were conducted in healthy adults. We excluded these studies during study selection. Two CTAR trials from the UK and India were registered in the International Clinical Trials Registry Platform of the World Health Organization (WHO) recently. Thus, research on the effects of CTAR exercise on post-stroke dysphagia is continuously increasing, and more high-quality RCTs from different countries are warranted to enrich our findings.

## Conclusion

In conclusion, the findings of this study suggest that CTAR exercise is an effective therapeutic method for post-stroke dysphagia rehabilitation and is superior to Shaker exercise in improving swallowing safety with positive effect to patients' psychological condition. Larger multicenter RCTs are needed to verify the effects of CTAR exercise in patients with different conditions, types of stroke, and times post stroke. It could also be worth to explore the optimal training dose of CTAR exercise to establish effective treatment protocols.

## Data availability statement

The raw data supporting the conclusions of this article will be made available by the authors, without undue reservation.

## Author contributions

JL and QW: screened the title, abstract, full-text of the identified studies, and drafted the manuscript. JT: performed the data extraction. WaZ and YiG: evaluated the risk bias of included studies. XC, WeZ, and YaG: performed the data verification. LZ: conceptualization, supervision, and funding acquisition. All authors reviewed and approved the final manuscript.
